# Random Forest Classification of Alcohol Use Disorder Using fMRI Functional Connectivity, Neuropsychological Functioning, and Impulsivity Measures

**DOI:** 10.3390/brainsci10020115

**Published:** 2020-02-20

**Authors:** Chella Kamarajan, Babak A. Ardekani, Ashwini K. Pandey, Sivan Kinreich, Gayathri Pandey, David B. Chorlian, Jacquelyn L. Meyers, Jian Zhang, Elaine Bermudez, Arthur T. Stimus, Bernice Porjesz

**Affiliations:** 1 Henri Begleiter Neurodynamics Lab, Department of Psychiatry, SUNY Downstate Health Sciences University, Brooklyn, NY 11203, USA; ashwini.pandey@downstate.edu (A.K.P.); sivan.kinreich@downstate.edu (S.K.); gayathri.pandey@downstate.edu (G.P.); david.chorlian@downstate.edu (D.B.C.); jacquelyn.meyers@downstate.edu (J.L.M.); jian.zhang@downstate.edu (J.Z.); arthur.stimus@downstate.edu (A.T.S.); bernice.porjesz@downstate.edu (B.P.); 2 Center for Biomedical Imaging and Neuromodulation, Nathan S. Kline Institute for Psychiatric Research, Orangeburg, NY 10962, USA; Babak.Ardekani@nki.rfmh.org; 3 Department of Psychiatry, NYU School of Medicine, New York, NY 10016, USA; bermue01@nyu.edu

**Keywords:** alcohol use disorder (AUD), functional connectivity, default mode network (DMN), resting state fMRI, neuropsychological performance, Tower of London Test, Visual Span Test, impulsivity, Random Forest

## Abstract

Individuals with alcohol use disorder (AUD) are known to manifest a variety of neurocognitive impairments that can be attributed to alterations in specific brain networks. The current study aims to identify specific features of brain connectivity, neuropsychological performance, and impulsivity traits that can classify adult males with AUD (*n* = 30) from healthy controls (CTL, *n* = 30) using the Random Forest (RF) classification method. The predictor variables were: (i) fMRI-based within-network functional connectivity (FC) of the Default Mode Network (DMN), (ii) neuropsychological scores from the Tower of London Test (TOLT), and the Visual Span Test (VST), and (iii) impulsivity factors from the Barratt Impulsiveness Scale (BIS). The RF model, with a classification accuracy of 76.67%, identified fourteen DMN connections, two neuropsychological variables (memory span and total correct scores of the forward condition of the VST), and all impulsivity factors as significantly important for classifying participants into either the AUD or CTL group. Specifically, the AUD group manifested hyperconnectivity across the bilateral anterior cingulate cortex and the prefrontal cortex as well as between the bilateral posterior cingulate cortex and the left inferior parietal lobule, while showing hypoconnectivity in long-range anterior–posterior and interhemispheric long-range connections. Individuals with AUD also showed poorer memory performance and increased impulsivity compared to CTL individuals. Furthermore, there were significant associations among FC, impulsivity, neuropsychological performance, and AUD status. These results confirm the previous findings that alterations in specific brain networks coupled with poor neuropsychological functioning and heightened impulsivity may characterize individuals with AUD, who can be efficiently identified using classification algorithms such as Random Forest.

## 1. Introduction

Alcohol use disorder (AUD) is a chronic, addictive, and relapsing disorder [1,2]. Individuals with chronic AUD manifest a variety of neurocognitive impairments [3], which may underlie both structural and functional features of the brain [4,5,6], and some of these impairments do not recover even after prolonged abstinence from drinking [7,8]. Recent studies have proposed the potential utility of resting state functional Magnetic Resonance Imaging (fMRI) connectivity as one of the neuroimaging biomarker for the quantitative clinical evaluation of AUD [9,10,11]. Therefore, it may be important to further confirm the utility of this neural measure as a potential biomarker, which can be used to improve the predictive accuracy of AUD diagnosis [11,12,13].

Recent studies are increasingly using Machine Learning (ML) approaches to predict and/or classify various neuropsychiatric disorders and outcomes [14,15,16], including AUD [11,17,18]. ML is becoming an essential part of data analytics [16], which can also handle numerous variables on a smaller sample size [19]. Random Forest (RF) is a widely used ML method to predict/classify individuals with a particular diagnosis from the unaffected controls [20]. RF uses randomly generated bootstrapped data sets that can then be used to train an ensemble of decision trees, which will determine an outcome by a majority “vote” to classify the data [21]. The main advantages of RF methods are: (i) they are non-parametric and therefore do not depend on the distribution of the data [20], they relatively have a smaller bias *and* less variance resulting in good generalization power [22], and (iii) they gracefully handle multi-collinearity in data, a problem that destabilizes traditional regression-based methods. 

Modern neuroscience views the brain as a complex organ with multiple, specialized, and interactive networks of distributed anatomical regions that support functions most basic to higher-order cognition [23,24,25]. Evidence from fMRI, electroencephalogram, (EEG), magnetoencephalogram (MEG), and other imaging studies also support the view that neurocognitive processing arise from coordinated networks of distributed brain areas [23,26,27,28] that mediate fundamental aspects of cognitive domains, including attention, perception, memory, language, and motor processing [29,30,31]. These large-scale brain networks also underlie cognitive and affective dysfunction in psychiatric and neurological disorders [32], including addiction [33]. Therefore, in order to understand the neurocognitive mechanism of alcohol addiction, it is vital to investigate functional integrity of the networks as well as neuropsychological functioning of individuals with AUD. At the behavior level, AUD is also characterized by increased impulsivity [34,35,36] and associated brain networks [10,37,38]. Therefore, it is desirable that any predictive model of AUD using brain network features should also include representative features from neurocognitive as well as impulsivity measures. 

Accumulating evidence from fMRI studies suggest that functional connectivity (FC), a measure to elicit temporal synchrony between neural signals across specific brain regions [39], is an effective method not only to study the functional organization of a healthy brain [40,41,42] but also to understand neurocognitive impairments and psychopathology [43,44]. Recent studies have also demonstrated that AUD is associated with atypical FC in resting state networks [9,10,11,45,46,47,48], thus compelling its use in ML models for predicting AUD. Among the resting state networks, the Default Mode Network (DMN) is the most studied network and has been shown to play a central role in the intrinsic network properties and neural organization during spontaneous mental processes [39]. Atypical DMN connectivity has been reported in alcohol and other substance use (see, review by Zhang and Volkow [49]), and the connectivity changes have been primarily attributed to craving and relapse in chronic abusers of drugs [49]. Aberrations in the DMN were shown to be related to impaired self-awareness, negative emotions, and to ruminations related to addiction [49].

The DMN, which is a functional network representing the ongoing mental processes during resting state, primarily involves basic neural activity underlying self-referential thought, mentation, and introspection [50], and recent studies have reported aberrant DMN connectivity in AUD [10,45,47]. In a study using fMRI FC to examine several resting state networks, including the DMN, Zhu et al. [11] reported that the RF method successfully classified AUD from control subjects both within and between networks. It is important to relate network dynamics to corresponding neuropsychological functions and behavioral outcomes, such as impulsivity, which are characteristic features in AUD [3,51,52]. For example, it is well-established that individuals with AUD manifest neuropsychological impairments in executive functioning, memory, and visuospatial processing [3,53,54,55,56], and studies have also demonstrated that AUD was associated heightened impulsivity [34,57]. Furthermore, all three domains are related among each other in the development and maintenance of alcohol addiction [58,59]. For instance, impulsivity observed in addicted individuals may result from a failure or dysfunction of the executive system and both domains may underlie distinct yet interactive brain systems [60]. Therefore, it is essential for the FC studies on AUD to include features from these domains (i.e., neuropsychological and impulsivity measures), which may have implications for diagnosis and/or preventive strategies. Therefore, the present study has been designed to perform a predictive model based on the RF classification of AUD using neural measures such as fMRI-based DMN FC together with relevant neuropsychological (i.e., executive functioning, and visual-spatial working memory) and personality (i.e., impulsivity) predictors. 

In this context, the goal of the current study is to identify specific features of FC, neuropsychological, and impulsivity measures that contribute to a successful classification of AUD individuals from healthy controls using an RF algorithm. Based on findings from previous studies, we also expect that the RF method will prove highly useful to successfully extract salient features from all three domains (FC, neuropsychological, and impulsivity) to classify AUD individuals from unaffected controls. Since individuals with chronic AUD are known to have deficits in all three domains that are related among themselves and with AUD, we hypothesize that specific connections across the DMN regions, especially the prefrontal–parietal and prefrontal–hippocampal connections, along with particular subsets of neuropsychological and impulsivity features will contribute to AUD classification as revealed by the importance rankings of the RF parameters. 

## 2. Methods

### 2.1. Participants

The demographic and clinical characteristics of the sample are presented in Table 1, and a detailed description is available in Pandey et al. [6]. All participants in the current study were drawn from the sample of a larger study on brain dysfunction in chronic alcoholism conducted at the SUNY Downstate Health Sciences University, Brooklyn, NY, USA. From a selected list of adult male participants who could be contacted during the MRI data collection period (*n* = 152), 68 individuals who had a lifetime AUD (DSM-IV alcohol dependence criteria) and 84 individuals who did not have any diagnosis of substance use disorders (SUD) were identified. Subjects from these subsets (AUD patients and non-SUD community controls) who agreed to participate and met the inclusion and exclusion criteria, including the MRI scanning protocols, were recruited for the current study until 30 subjects in each group (AUD and controls) were successfully scanned. Thus, the final sample comprised thirty male participants with AUD (mean age (SD) = 41.42 (7.31) years) and thirty unaffected male controls (mean age (SD) = 27.44 (4.74) years). The race distribution of the sample was: Black/African American = 25; White/European American = 9; Asian = 21; American Indian = 1; More than one race = 2; and Unknown = 2. Participants with AUD were recruited from alcohol treatment centers in and around New York City after they had been detoxified and were abstinent for at least 30 days prior to testing. Some of the participants from the AUD group had consumed tobacco (*n* = 20) and/or marijuana (*n* = 10) during the last 6 months (but not at least 5 days before testing). None of the participants were in withdrawal for alcohol or any other drugs (including for nicotine) at the time of testing. Individuals for the control group (CTL) were recruited through advertisements and screened to exclude any personal or family history of major medical, psychiatric, or substance-related disorders. None of the participants from the control group ever met the diagnosis of substance dependence or abuse (DSM-IV), although some of the control participants (*n* = 12) were light/regular drinkers and had used alcohol in the last 6 months (*n* = 18) (see Table 1 for details). All participants were asked to abstain from alcohol and other drugs for 5 days prior to MRI scans. A modified version of the semi-structured assessment of genetics of alcoholism (SSAGA) [61] was administered to assess alcohol/substance use and related co-existing disorders and family history of these disorders. The majority of subjects were right-handed, with only a few who were either left-handed (5 in the AUD group and 2 in the CTL group) or bi-dexterous (2 in the AUD group and 1 in the CTL group). Clinical and psychometric data were collected at the SUNY Downstate Health Sciences University, while the fMRI data were acquired at the Nathan Kline Institute (NKI) for Psychiatric Research. Standard MRI protocols and exclusion criteria (implants, tattoos, cosmetics, claustrophobia, etc.) were used to ensure subjects’ safety and the quality of data. Individuals with hearing/visual impairment, a history of head injury or moderate and severe cognitive deficits (>21) on the mini-mental state examination (MMSE) [62] were also excluded from the study. Informed consent was obtained from the participants and the research protocol was approved by the Institutional Review Boards of both centers (IRB approval ID: SUNY–266893; NKI–212263).

### 2.2. Neuropsychological Assessment

#### 2.2.1. Tower of London Test (TOLT)

Computerized adaptations of the Tower of London Test (TOLT) [63] and the Visual Span Test (VST) [64,65] were administered using the Colorado assessment tests for cognitive and neuropsychological assessment [66], as described previously [6]. Planning and problem-solving ability of the executive functions were assessed using the TOLT in which participants solved a set of puzzles with graded difficulty levels by arranging the color beads one at a time from a starting position to a desired goal position in as few moves as possible. The test consisted of 3 puzzle types with 3, 4, and 5 colored beads placed on the same number of pegs, with 7 problems/trials per type and a total of 21 trials. Five performance measures from the sum total of all puzzle types were used in the analysis: (i) excess moves (additional moves beyond the minimum moves required to solve the puzzle); (ii) the average pickup time (initial thinking/planning time spent until picking up the first bead to solve the puzzle); (iii) the average total time (total thinking/planning time to solve the problem in each puzzle type); (iv) the total trial time (total performance/execution time spent on all trials within each puzzle type); and (v) the average trial time (mean performance/execution time across trials per puzzle type).

#### 2.2.2. Visual Span Test

The VST was used to assess visuospatial memory span from the forward condition and working memory from the backward condition. In this test, 8 randomly arranged squares were displayed on the screen, and 2–8 squares flashed in a predetermined sequence depending on the span level being assessed. Each span level was administered twice, with a total of 14 trials in each condition. During the forward condition, subjects were required to repeat the sequence in the same order via mouse clicks on the squares. In the backward condition, subjects were required to repeat the sequence in reverse order (starting from the last square). Four performance measures were collected during forward and backward conditions (with a total of 8 scores): (i) the total correct trials (total number of correctly performed trials); (ii) the span (maximum sequence-length achieved); (iii) the total average time (sum of the mean time taken across all trials performed); and (iv) the total correct average time (sum of the mean time taken across all trials correctly performed).

### 2.3. Impulsivity Scores

Impulsivity was assessed using the Barratt Impulsiveness Scale—Version 11 (BIS-11) [67], which is a 30-item self-administered tool with excellent psychometric properties [68]. The impulsivity scores consisted of three factors: motor impulsivity (BIS_MI), non-planning (BIS_NP), and attentional impulsivity (BIS_AI), and a total score (BIS_Tot).

### 2.4. MRI Data Acquisition

MRI scans were acquired at the Nathan Kline Institute using a 3.0 Tesla Siemens Tim Trio scanner (Erlangen, Germany). A continuous resting-state fMRI scan was acquired for the duration of 6.11 min in both AUD and CTL participants, who were instructed to keep their eyes closed but to stay awake and not to move. A series of T2*-weighted gradient echo single-shot *echo-planar imaging* (EPI) volumes with the following sequence parameters was acquired: Repetition Time (TR) = 2750 ms; Echo Time (TE) = 30 ms; flip angle = 80°; voxel size = (2.5 × 2.5 × 3.5) mm^3^; matrix size = 96 × 96; number of slices = 34; number of volumes = 130; Field of View (FOV) = 240 mm; and Grappa acceleration factor = 3. The sequence was carefully optimized to minimize the effects of magnetic susceptibility inhomogeneities (such as distortions and signal dropouts), as well as the effects of mechanical vibrations, which elevate Nyquist ghosting levels. In addition, a *magnetization-prepared rapid gradient-echo* (MPRAGE) high-resolution three-dimensional T1-weighted structural image was also collected to be used as an anatomical reference for the fMRI data and for the non-linear registration of imaging data between subjects. The sequence parameters for the MPRAGE were: TR = 2500 ms; TE = 3.5 ms; Inverse Time (TI) = 1200 ms; flip angle = 8°; voxel size = 1 × 1 × 1 mm^3^; matrix size = 256 × 256 × 192; FOV = 256 mm; and number of averages = 1.

### 2.5. Image Processing

Processing of the imaging data included the following stages. Within each subject, the MPRAGE and fMRI volumes were registered using the intra-subject inter-modality linear registration module [69] of the automatic registration toolbox (ART; https://www.nitrc.org/projects/art). The *brainwash* program within the ART toolbox was used for skull-stripping the MPRAGE volumes. To correct for small subject motion during fMRI acquisitions, motion detection and correction was performed using the *3dvolreg* module of the AFNI software package [70]. To correct for the geometric distortions of the fMRI images due to magnetic susceptibility differences in the head, particularly at brain/air interfaces, we used the non-linear registration module of the ART [71]. The skull-stripped MPRAGE images from all subjects were non-linearly registered to a study-specific population template using ART’s non-linear registration algorithm, which is one of the most accurate inter-subject registration methods available [72]. The population template was formed using an iterative method [73]. The motion corrected fMRI time-series were detrended using PCA [74]. Finally, fMRI from all subjects were normalized to a standard space using the image registration steps outlined above, which were mathematically combined into a single transformation and used in re-sampling the fMRI. 

### 2.6. DMN Seed Regions and FC Calculations

**Figure 1:**
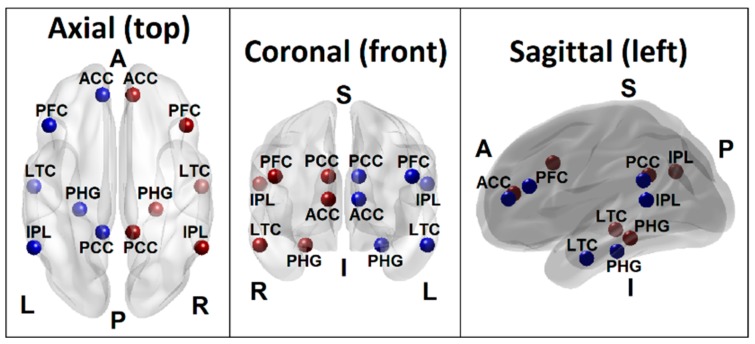
Seed regions of the DMN consisting of 6 regions in the left hemisphere (blue beads) and 6 homologous regions in the right hemisphere (red beads), as listed in Table 1. Axial (top), coronal (front), and sagittal (left side) views are shown. (PCC–Posterior cingulate cortex; ACC–Anterior cingulate cortex; IPL–Inferior parietal lobule; PFC–Prefrontal cortex; LTC–Lateral temporal cortex; PHG–Parahippocampal gyrus; L–Left; R–Right; A–Anterior; P–Posterior; S–Superior; I–Inferior).

Based on the empirical evidence from extensive rs-FC fMRI literature, the recent conceptualization of the default mode network by Andrews-Hanna et al. [75] has included voxels spanning six anatomical regions: (i) the medial prefrontal cortex (mPFC; dmPFC, rostral ACC, and parts of the anterior and ventral mPFC), (ii) the lateral frontal cortex (superior frontal cortex and inferior frontal gyrus), (iii) the medial parietal cortex (PCC and retrosplenial cortex), (iv) the medial temporal lobe (hippocampus and PHF), (v) the lateral parietal cortex (spanning angular gyrus and posterior supramarginal gyrus), and (iv) the lateral temporal cortex (LTC) (extending anteriorly to the temporal poles). The seed regions of the DMN for the current study were adapted from this theoretical review as well as from the LORETA (Low Resolution Electromagnetic Tomography) studies on EEG current sources by Imperatori et al. [76] and Thatcher et al. [77] who have reportedly derived the seeds from the work of Buckner et al. [78]. These seed regions included six anatomical locations involving both hemispheres: the posterior cingulate cortex (PCC), anterior cingulate cortex (ACC), inferior parietal cortex (IPL), PFC, LTC, and the parahippocampal gyrus (PHG) (Table 2 and Figure 1). Each seed region contained the voxels within a 10 mm radius from the peak/centroid point of the anatomical locations referenced using the eLORETA software [79]. The ROI-to-ROI connectivity [80], the most commonly used method to derive FC across brain regions [81], was computed using Pearson correlation coefficients between all unique pairs (*N* = 66) of BOLD time series data of the DMN seed regions listed in Table 2. The resulting correlation coefficients were Fisher Z-transformed for further statistical analyses. 

### 2.7. Random Forest Classification Model and Parameters

Random forest classification analysis was performed using R-packages “randomForest” (https://cran.r-project.org/web/packages/randomForest), “caret” (https://cran.r-project.org/web/packages/caret), and “randomForestExplainer” (https://cran.r-project.org/web/packages/randomForestExplainer). A Random Forest classifier consists of a collection of tree-structured classifiers where each tree casts a unit vote for a class/group for each set of predictor variables [82]. A growing number of studies in computational biology are using RF because (i) it is nonparametric, interpretable, efficient, and (ii) it has high prediction accuracy for many types of data due to its unique advantages in dealing with a small sample size, high-dimensional feature space, and complex data structures [83]. The two main parameters of the random forest algorithm are the number of trees in the ensemble and the number of variables randomly selected for the splitting decision at each node. Two levels of randomness are used by the random forest to construct the ensemble of trees: first, the model trains itself using a training data for creating each tree based on bootstrap aggregating (bagging). At the second level, the algorithm randomly selects a subset of features to split at each node while growing a decision tree for group classification. In order to maximize the classification accuracy (by reducing the errors or impurity), only a single best feature (variable) among a random subset of features is selected at each internal node. This process is recursively repeated until one of the three conditions is met: (i) the tree has either reached a specified depth, (ii) the number of samples in a node becomes lower than the set threshold, and (iii) when all the samples are grouped into the same category [84]. Some of the important concepts and parameters of the Random Forest classification method are listed in Table A1 (see Appendix A).

The Random Forest classification model included 66 DMN connections, 13 neuropsychological scores, and 4 BIS scores as features, while the group status (AUD and CTL) served as the outcome variable. The training data consisted of a full sample for identifying significant features for classifying the groups. To compute prediction error and classification accuracy, we used the Out-of-Bag (OOB) error method. According to Breiman and Cutler [85], in random forests, there is no need for cross-validation or a separate test sample to get an unbiased estimate of the test sample error, which is estimated internally in the algorithm. Each decision/classification tree is constructed using a different bootstrap sample from the training data (due to random selection), and about one-third of the observations from the training data are left out during each bootstrap, called the out-of-bag sample, which will be used only to estimate the prediction accuracy of the RF model. While classification trees are grown for each bootstrap sample (which is approximately two-thirds of the training data), the OOB error rate is calculated for each classification tree being built. The aggregate of OOB scores on all ‘ntree’ trees (which is the maximum number of trees preset in the model calculation) will provide the ensemble OOB error rate. Thus, the OOB score provides a validation for the RF model. In the model used in the current study, the maximum number of trees ‘ntree’ was set at 500 (default). The optimal number of features analyzed at each node (‘Mtry’) was estimated to be 10 (using the “tuneRF” function) and was used in the classifier algorithm. The final list of variables that significantly contributed for the classification was tabulated, and 3-dimensional connectivity maps of top significant DMN connections within a brain anatomical template were created using custom Matlab scripts. 

## 3. Results

### 3.1. Random Forest Classification

#### 3.1.1. Classification Accuracy and Top (Ranked) Significant Variables

**Figure 2:**
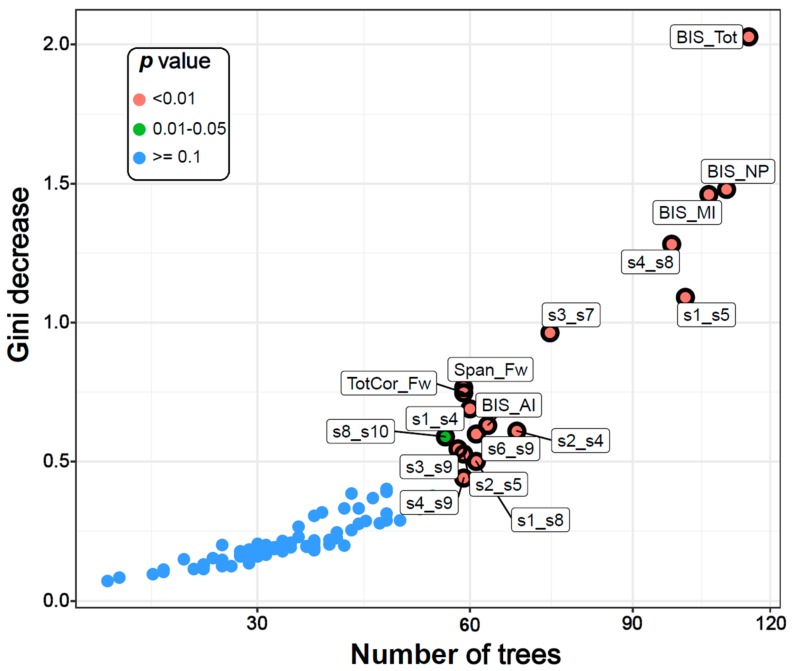
The multi-way importance plot showing the top significant variables (labelled and marked with black circles) that contributed to the classification of AUD from CTL individuals based on the following measures: the Gini decrease, the number of trees, and the *p*-values. Fourteen DMN connections, two neuropsychological variables, and all four impulsivity scores were significant (circled and labelled red and green dots). BIS–Barratt Impulsivity Scale; MI–Motor impulsivity; NP–Non-planning; AI–Attentional impulsivity; Tot–Total; Span_Fw–Span forward; TotCor_Fw–Total correct forward; s1-s12–DMN seeds 1-12 as listed in Table 2.

**Figure 3:**
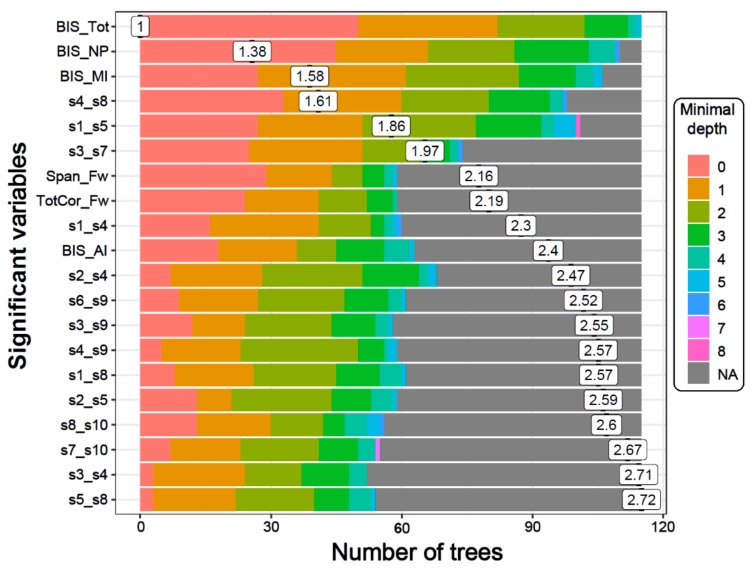
The distribution of minimal depth among the trees of the forest for the significant variables is shown in different colors for each level of minimal depth. The mean minimal depth in the distribution for each variable is marked by a vertical black bar overlapped by its value inside a box. A lower mean minimal depth of a functional connectivity (FC) variable represents a higher number of observations (participants) categorized in a specific group on the basis of the variable. The top significant variables (14 DMN connections, two neuropsychological scores, and all four BIS scores) followed the same rank in the plot as in Table 3, which is ordered based on *p*-values.

**Figure 4:**
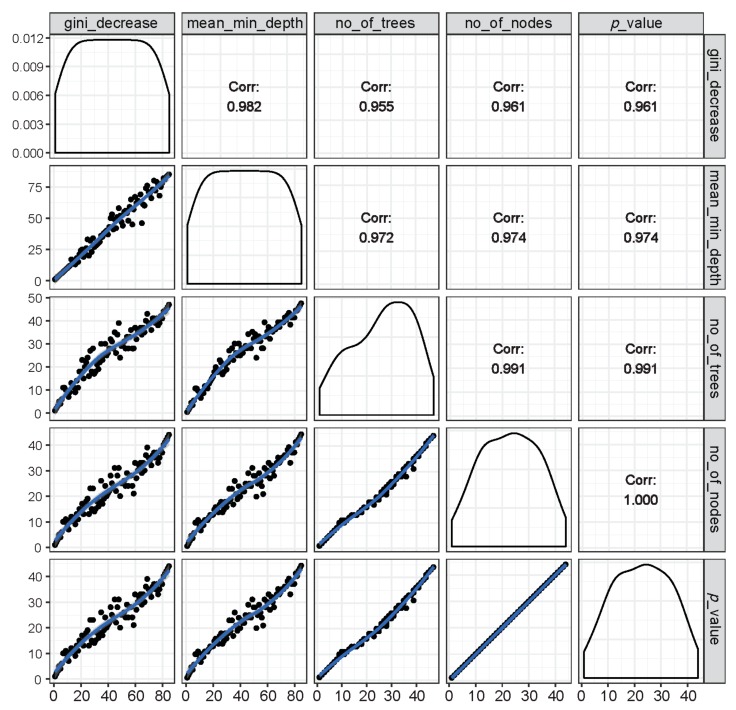
Illustration of rankings of variables based on any of the two RF parameters of importance (inside the left and bottom panels showing distribution of the rankings of all predictor variables marked with black dots along a blue trend line) as well as the respective correlation coefficients across rankings of any two parameters (inside the right and top panels). It is shown that all RF parameters of importance were found to have very high correlations among each other, suggesting that these parameters are highly reliable at ranking the importance of variables in group classification.

The classification accuracy was 76.67% as the RF algorithm correctly classified 23 out of 30 subjects in each group. The model identified 14 DMN connections, two neuropsychological variables (memory span and total correct scores of the forward condition of the VST), and all four impulsivity scores as significantly contributing to classifying individuals into either the AUD or CTL group (Table 3 and Figure 2 and Figure 3). Among the significant DMN connections, the AUD group showed hyperconnectivity in three DMN connections across bilateral ACC and bilateral PFC (L.ACC–L.PFC, L.ACC–R.ACC, and R.ACC–R.PFC) as well as in two connections across bilateral PCC and left IPL (L.PCC–L.IPL and R.PCC–L.IPL), while showing hypoconnectivity in rest of the connections across the anterior–posterior and interhemispheric regions (L.PCC–R.ACC, R.PCC–R.ACC, L.PCC–R.PFC, L.ACC–L.LTC, R.ACC–L.LTC, L.PFC–R.LTC, L.IPL–R.PFC, R.IPL–L.LTC, and R.PFC–R.LTC) (see Figure 4 and Table 3). AUD individuals also showed relatively poorer visuo-spatial memory performance and higher impulsivity than controls.

#### 3.1.2. Multi-Way Importance Plot

The top significant variables were also shown in a multi-way importance plot based on the relationships across three RF measures of importance, viz., *the Gini decrease*, *the number of trees*, and *the p-value* (Figure 2). A particular variable is deemed significant if that variable is used for splitting more often than that of a random chance. As listed in Table 3, the variables that were found to be important for group classification are: 14 DMN connections, two neuropsychological variables (the memory span and total correct scores of the forward condition of the VST), and all four impulsivity (BIS) scores. 

#### 3.1.3. Distribution of Minimal Depth

The distribution of minimal depth among the trees of the forest for the top significant variables is shown in Figure 3. The minimal depth of a variable represents the depth of the node that splits on that variable and is the closest to the root of the decision tree. A lower mean minimal depth of a variable represents a higher number of observations (participants) categorized in a specific group on the basis of that variable. The order/rank of the top significant variables (14 DMN connections, two neuropsychological scores, and all four impulsivity scores) followed the same pattern in the minimal depth plot, which is based on the minimal depth and the number of trees. 

#### 3.1.4. Relations among Rankings of Different RF Parameters

The relations among rankings of different RF parameters are shown in Figure 4. The correlations across any two parameters were very high, suggesting that the importance rankings of variables based on different RF parameters were mostly similar and that these parameters are highly reliable and consistent in the classification performance. 

#### 3.1.5. Connectivity Mapping of Significant DMN Connections

**Figure 5:**
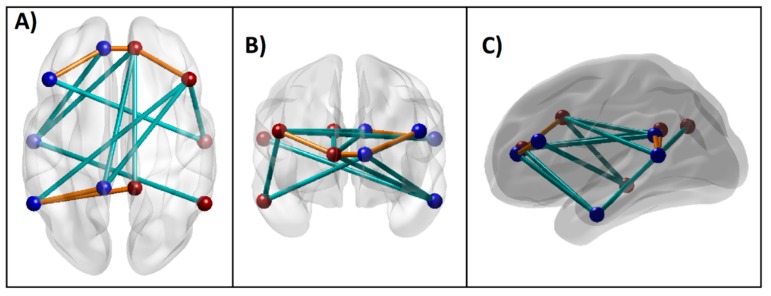
Significant DMN connections that contributed to the Random Forest classification of AUD from CTL individuals based on importance parameters including the *p*-value (*p* < 0.01), as listed in Table 3. These connections (i.e., edges) across the seed regions (i.e., nodes) within an anatomical brain template are shown: (**A**) axial (top) view; (**B**) coronal (front) view; and (**C**) sagittal (left side) view. The blue and red beads represent left and right-sided nodes, respectively, and the edges in orange and cyan lines represent hyper- and hypo-connectivity, respectively, in the AUD compared to the CTL group.

The 3-D brain connectivity map of significant DMN connections, which contributed to group classification, is shown in Figure 5. Compared to the CTL group, AUD individuals showed hyperconnectivity in five of the significant DMN connections (orange lines), while showing hypoconnectivity in the remaining nine connections (cyan lines). Specifically, the AUD group showed hyperconnectivity across bilateral ACC and PFC as well as between bilateral PCC and left IPL, while showing hypoconnectivity across other regions primarily involving anterior–posterior and interhemispheric long-range connections.

### 3.2. Correlations between Top Significant Variables and Age

Since age difference across the groups was statistically significant (*p* < 0.001), the association of age with significant predictor variables was evaluated within each group using the bivariate Pearson correlation as well as in the total sample using partial correlation adjusted for group effect (Table 4). Overall, age was not found to have robust effects on most of the top variables. On the other hand, age was negatively correlated (*p* < 0.05) with the top two neuropsychological variables (memory span and total correct scores of the forward condition of the VST) only within the AUD group, suggesting that older AUD individuals displayed poorer memory performance than younger AUD subjects. Furthermore, age was negatively correlated (*p* < 0.05) with the L.ACC–R.ACC connection in the CTL group while it was positively correlated (*p* < 0.05) with the R.IPL–L.LTC connection in the total sample.

### 3.3. Correlations among the Top Significant Variables

**Figure 6:**
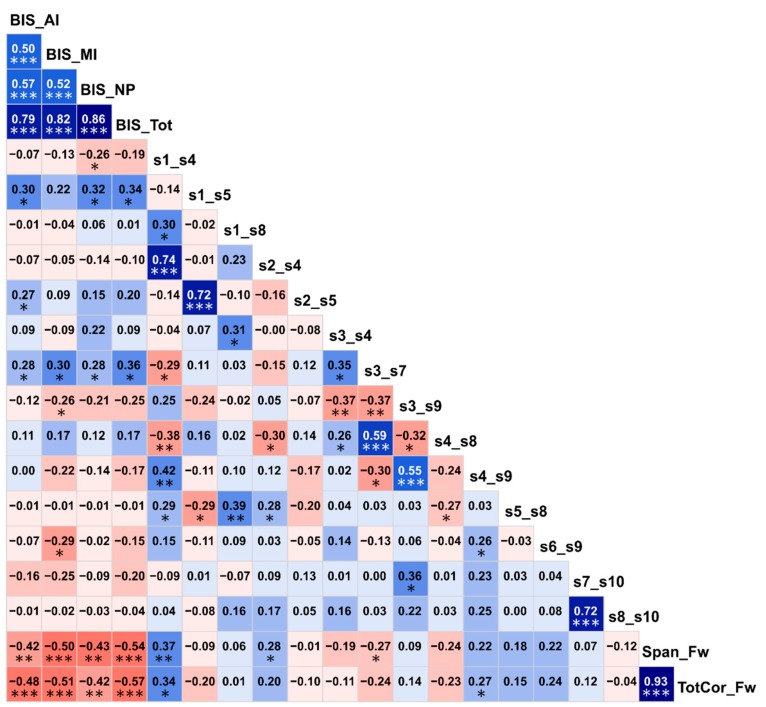
Correlation matrix showing associations among the top significant variables. The values within each cell represent the bivariate Pearson correlation between the variable on its vertical axis and the variable on its horizontal axis. The correlation values are color coded (red/pink shades represent negative r-values, blue/cyan shades indicate positive r-values, darker shades represent relatively higher magnitudes) and significant correlations have been marked with asterisks (*p < 0.05; **p < 0.01; and ***p < 0.001).

Correlations among the top significant variables are shown in Figure 6. It was found that BIS impulsivity scores had highly significant negative correlations (*p* < 0.01) with visual memory performance (i.e., memory span and total correct scores of the forward condition of the VST), indicating that individuals with higher impulsivity showed poorer visual memory performance. Interestingly, of the six FC variables that showed significant correlations (*p* < 0.05) with one or more BIS scores, three DMN connections (s1–s5/L.PCC–L.IPL, s2–s5/R.PCC–L.IPL, and s3–s7/L.ACC–L.PFC) were positively correlated with impulsivity as well as having hyperconnectivity in AUD (Figure 5), whereas the other three DMN connections (s1–s4/L.PCC–R.ACC, s3–s9/L.ACC–L.LTC, and s6–s9/R.IPL–L.LTC) were negatively correlated with impulsivity (*p* < 0.05) as well as having hypoconnectivity in AUD (Figure 5), thus linking altered FC and higher impulsivity with AUD status. Furthermore, of the four FC variables that had significant correlations with two visual memory scores (i.e., the memory span and total correct scores of the forward condition of the VST), three of them (s1–s4/L.PCC–R.ACC, s2–s4/R.PCC–R.ACC, and s4–s9/R.ACC–L.LTC) showed significant positive correlations (*p* = 0.05–0.01) and one of the connections (s3–s7/L.ACC–L.PFC) showed a significant negative correlation (*p* < 0.05) with visual memory scores. Interestingly, the three connections that had positive correlations with visual memory scores also had hypoconnectivity in AUD (Figure 5), whereas the single negatively correlated connection had hyperconnectivity in AUD (Figure 5), thus linking altered FC and poor neuropsychological performance with AUD status. Although similar correlations were observed within each group, these correlations were either less significant or non-significant in the within-group analysis possibly due to a relatively smaller sample size (*N* = 30).

## 4. Discussion

The current study aimed to identify specific features of FC, neuropsychological, and impulsivity to classify individuals with AUD from a CTL group. Findings showed that the RF model achieved a classification accuracy of 76.67% and identified 14 DMN connections, two neuropsychological variables (memory span and total correct scores of the forward condition of the VST), and all impulsivity factors as important features to classify participants into either AUD or CTL group (Table 3 and Figure 2, Figure 3 and Figure 5). Relative to the CTL group, AUD individuals manifested hyperconnectivity across the bilateral anterior cingulate cortex (ACC) and prefrontal cortex (PFC) as well as between the bilateral posterior cingulate cortex (PCC) and the left inferior parietal lobule (IPL), while showing hypoconnectivity in several connections involving anterior–posterior and interhemispheric long-range connectivity (Figure 5). Furthermore, AUD subjects showed poorer neuropsychological performance and visual spatial memory span as well as increased impulsivity compared to the CTL group. Furthermore, the top important variables from the three different domains, as identified by the RF method, were also associated with each other as well as with AUD status (Figure 6).

### 4.1. Aberrant FC in Individuals with AUD

#### 4.1.1. Hyperconnectivity within Frontal and Parietal Regions in AUD

The observed hyperconnectivity across prefrontal regions in AUD is an important finding, given the prominent role of ACC and PFC in human cognition [86,87,88,89] as well as in the development of AUD [4,90,91,92,93]. Therefore, it is highly likely that altered FC within prefrontal DMN nodes may be a manifestation of these structural/functional impairments of frontal lobe regions in AUD. Previous findings from the literature also suggest that prefrontal hyperconnectivity in AUD may have resulted in increased impulsivity and a lack of inhibitory control in these subjects, as there is an association between prefrontal hyperconnectivity and externalizing traits, such as impulsivity, aggression, psychopathy, [94,95,96] and ADHD [97,98]. Therefore, the observed hyperconnectivity across the prefrontal regions may indicate a state of neural hyper-excitability in individuals with AUD [99]. In the same groups of subjects, we have recently reported that the AUD group had smaller volumes in frontal cortices (left pars orbitalis, right medial orbitofrontal, right caudal middle frontal regions) as well as in bilateral hippocampi [6], further confirming possible aberrations frontal networks exhibited by AUD patients. Furthermore, the increased impulsivity and visual memory performance observed in the current study could also be related to hyperconnectivity in the frontal networks observed in the current study as well as to the structural deficits reported in our earlier work on the same participants [6]. On the other hand, the AUD group also manifested local hyperconnectivity within the posterior regions (bilateral PCC–L.IPL). It is well known that PCC and IPL are pivotal regions for social cognition [100], and recent studies have suggested that hyperconnectivity between these regions may indicate interpersonal and emotional disturbances [101,102]. During a cue-reactivity performance in AUD patients (without a control group), Huang et al. [103] observed hyperconnectivity between dorso-medial PFC and insula anteriorly and between PHG and angular gyrus posteriorly in addition to long-range hypoconnectivity across multiple regions during the alcoholic beverage condition compared to non-alcoholic beverage condition. Taken together, local hyperconnectivity in frontal and parietal regions may indicate aberrations in emotional, motivational and social behaviors, possibly giving rise to externalizing features in AUD. However, since prior FC findings in AUD are limited, more DMN studies in different subgroups of AUD are warranted in order to further confirm and understand the present findings.

#### 4.1.2. Hypoconnectivity across Anterior–Posterior and Interhemispheric Connections in AUD

In tandem with the local hyper-connectivity in prefrontal and parietal regions (as discussed in the previous section), the AUD group also manifested hypoconnectivity in the majority of the top significant connections (9 of 14), which included anterior–posterior and interhemispheric long-range connections (Figure 5) across fronto-parietal and fronto-temporal regions. While the fronto-parietal network includes hub regions for cognitive control [104] and is implicated in several neuropsychiatric disorders [105], the fronto-temporal network subserves linguistic processing [106] and social cognition [107] and is primarily implicated in psychoses and autism [108,109,110]. As mentioned above, there are only a handful of fMRI FC studies on AUD and they differ extensively in methodology and the networks that were examined. On the other hand, a few studies on other addictions point to converging findings. For example, a recent study on internet gaming addiction suggested that the addicted individuals showed hypoconnectivity in the long-range anterior–posterior connections, viz., between the medial PFC and the PCC and between the left IPL and the medial PFC [111]. In a study on smoking addiction, Tang et al. [112] reported that smokers had reduced effective connectivity from the PCC to the medial PFC (ACC) and from the IPL to the medial PFC, compared to non-smokers. As mentioned earlier, Huang et al. [103] reported hypoconnectivity in long-range connections across multiple regions of reward and executive processes in AUD patients with excessive craving, in addition to local hyperconnectivity at anterior and posterior regions, while processing alcohol cues. These studies from drug and behavioral addictions suggest that there might be a common substrate for the entire addiction spectrum disorders, which can include addiction for substances and behaviors. On the other hand, hyperconnectivity within prefrontal nodes observed in the current study may be interpreted as a compensatory mechanism to overcome the functional loss due to prefrontal hypoactivity found in abstinent AUD individuals [113]. Furthermore, diffusion tensor imaging (DTI) studies have reported widespread abnormalities in the white matter tracts of associative fibers [114,115] as well as interhemispheric fibers [6,116]. In sum, although these findings support the view that weaker anterior–posterior connectivity representing attentional and higher-cognitive processing can be a marker in addiction in general, more FC studies on AUD and related disorders are needed to elucidate distinct and shared neural mechanisms underlying AUD and other addictions.

### 4.2. Poor Neuropsychological Performance in AUD

The RF model also identified two scores in the VST forward condition as important features to classify those with AUD from controls: (i) memory span, and (ii) total correct score. The AUD participants showed poor performance in terms of lower memory span and less correct trials compared to CTL participants. It has long been established that individuals with AUD manifest neuropsychological impairments in multiple domains, such as deficits in executive functioning, memory, and visuospatial processing [3,53,54,55,56,117]. It is also known that while the recovery of some cognitive processes are known to occur, certain deficits can persist even after prolonged abstinence [8]. In our sample of abstinent AUD individuals, the observed visuospatial memory deficits despite the abstinent status, suggests that residual cognitive deficits can potentially impair neural processes needed to encode and maintain stimulus sequences, such as rehearsal. These impairments may also interfere with higher cognitive processes during task performance or real-life functioning that involve these visual memory processes. In our previous study on the same groups of subjects, we reported that the AUD group had a smaller bilateral hippocampal volume [6] and that lower volumes in prefrontal cortex and left hippocampus were associated with poorer visuospatial memory performance [6]. Furthermore, the hyperconnectivity across parahippocampal hub in the current study may also be related to the visual memory deficit, possibly representing a compensatory mechanism during the memory performance. On the other hand, in the context of numerous reports of impaired executive functioning in AUD [8], it was surprising to find that the TOLT variables failed to emerge as important features for classification. It is possible that the AUD sample in our study, due to their abstinent status, manifested significant deficits mainly in visual short-term memory in the VST but the executive functioning as tapped by the TOLT was either largely recovered due to abstinence. Furthermore, it is also possible that TOLT scores might be more relevant in the RF model involving FC features of other networks during resting state (e.g., executive control, attentional, etc.) or task performance (e.g., response inhibition, reward processing, etc.) rather than the DMN as used in the current study. Future FC studies during resting state as well as task performance using various domains of neuropsychological features may resolve this puzzle.

### 4.3. Heightened Impulsivity in AUD

Our finding shows that all BIS impulsivity scores were among the top variables that classified individuals with AUD from the CTL group. The AUD group also showed significantly increased impulsivity compared to the controls. This finding supports the existing view that impulsivity is a core feature of substance use disorders and may result from impaired inhibitory control [35]. We have also observed that six of the top FC variables showed significant correlations with one or more BIS scores (Figure 6). These findings can be supported by earlier studies that have drawn etiological connections among AUD, externalizing traits such as impulsivity, and neural disinhibition in the form of electrophysiological features (e.g., low P3 amplitude and delta and theta oscillations underlying P3 during cognitive processing, and increased resting state beta power) [34,99,118,119]. Importantly, impulsivity was found to be associated with reduced P3 amplitude in AUD [34] and other externalizing disorders [120,121,122]. The heightened impulsivity observed in the AUD group may also be related to altered FC at the frontal connections in the current study and to the lower volumes observed in frontal regions in the same subjects, as reported in our previous study [6]. There is also evidence from other studies that both structural and functional aspects of frontal lobes contribute to increased impulsivity in AUD patients [38,58,123,124]. Furthermore, recent studies have found association of impulsivity with resting state measures of EEG power [125], EEG-based FC [126] and fMRI-based FC [10], suggesting that impulsivity in AUD may underlie specific brain networks. 

### 4.4. Associations among AUD, FC, Impulsivity, and Neurocognition

The observed correlations among FC, neuropsychological performance, and impulsivity measures may indicate reciprocal interactions across these domains (Figure 6). It is interesting to note that increased impulsivity and poor neuropsychological performance were also associated with altered FC in AUD (e.g., hyper-connectivity was associated with increased impulsivity, and FC connections that had positive correlations with visual memory scores also showed hypoconnectivity in AUD), thus linking all three domains with AUD status as well. Although previous studies have separately shown that AUD was associated with altered FC [10,126], poor neuropsychological performance [3,54], and heightened impulsivity [34,57], no previous studies have incorporated all three domains in a single study, making the current study the first to examine these domains together. In our previous study on the same participants, we showed that lower volumes in frontal regions and hippocampus were associated with poorer neuropsychological functioning in AUD subjects [6], and studies are underway in our laboratory to incorporate structural and functional brain measures as well as neuropsychological and behavioral (e.g., impulsivity) in the predictive model of AUD. Future studies may also include comprehensive measures in each domain and implement predictive and path models to understand the exact nature of these associations. 

### 4.5. Potential Implications, Limitations, and Future Directions

The findings of the present study have shown that features from all three domains have contributed to the classification of AUD from CTL individuals, as shown in the list of important variables identified by the RF model (see Table 2 and Figure 2 and Figure 3). The implications of these findings are the identification of potential biomarkers of alcoholism, which may include a combination of altered DMN FC (local hyperconnectivity at the frontal and parietal regions and long-range hypoconnectivity across the anterior–posterior and interhemispheric connections), coupled with poor neuropsychological performance and increased impulsivity. These findings may aid in designing intervention programs for AUD. Nevertheless, our study has several limitations that future studies may aim to overcome: First, the age difference between AUD and CTL groups was significant as the groups were not matched for age. Despite this limitation on the age difference, the findings may still be valid as there were no systematic correlations across age and the top significant variables. However, we do acknowledge that age matching across the groups may be crucial as functional connectivity changes due to a neuropsychiatric condition, as in the case of autism spectrum disorders, may show opposing patterns during different developmental stages [127]. Alternatively, future studies may consider adding demographic factors (i.e., age, gender, race, education, etc.) in the classification model in order to determine the predictive significance of these factors. Second, the sample of the current study contained only males and therefore the generalizability of the findings may be limited. It is suggested that future studies use both age- and gender-matched groups. Third, the influence of a family history of AUD was not considered in the current study, and future studies may additionally examine family history information in order to explore whether the aberrations in FC are due to chronic alcohol consumption or preexisting neural endophenotypic features. Fourth, the range of neuropsychological testing was limited to only two tests, and would be strengthened by future studies that administered a comprehensive battery of neuropsychological tests to elucidate specific patterns of deficits and their interactions across different domains. Fifth, the current study has used a single measure (BIS) of impulsivity, while the inclusion of other related traits (e.g., sensation seeking, delay discounting, etc.) may shed more light on the etiological connections among these variables in the development and/or maintenance of AUD. Sixth, studies may explore a range of disorders in the addiction spectrum, which can include both substance and behavioral addiction, in order to identify common neural substrates underlying the spectrum as well as distinct effects of specific substance or behavior. Finally, multimodal measures of brain connectivity, including structural MRI (white matter fiber tracts) and EEG measures, may help our understanding of the complex relationships between structural and functional brain connectivity [128] and their role in AUD, and these studies are underway in our lab. More synthesis across various brain imaging methods will be essential not only for cross-validating the findings across different modalities but also for interpreting them in the light of one another.

## Figures and Tables

**Table 1:** Demographic and clinical characteristics of the sample.

Variable	AUD			CTL		
	*n* *	Mean	SD	*n* *	Mean	SD
Age (in years)	30	41.42	7.31	30	27.44	4.74
Education (in Years)	30	11.93	2.35	30	15.77	1.87
Age of onset (regular alcohol use)	30	15.77	2.58	12	20.50	3.80
Alcohol: Drinks/day (heavy alcohol use period)	30	12.08	10.02	12	2.88	1.93
Alcohol: Days/month (heavy alcohol use period)	30	20.30	9.01	12	3.35	3.64
Alcohol: Drinks (last 6 months)	30	2.68	6.61	18	2.61	1.98
Alcohol: Days (last 6 months)	30	3.97	8.02	18	2.94	3.62
Length of Abstinence (in months)	30	22.43	28.16	18	1.9	4.99
Tobacco: Times/day (last 6 months)	20	9.90	5.80	6	2.33	1.63
Tobacco: Days/month (last 6 months)	20	28.35	4.83	6	14.17	13.82
Marijuana: Times in last 6 months	10	98.80	91.38	4	18.75	27.61

* *n* refers to the number of subjects included in these mean and SD calculations for each variable. Individuals who did not consume alcohol or drugs were not included in the respective calculations.

**Table 2:** Default Mode Network (DMN) seed regions and the Montreal Neurological Institute (MNI) coordinates, as referenced from the exact Low Resolution Electromagnetic Tomography (eLORETA) software.

Seed	Region Name	Region Code	BA	MNI (X)	MNI (Y)	MNI (Z)
**s1**	Left posterior cingulate cortex	L.PCC	23	−10	−45	25
**s2**	Right posterior cingulate cortex	R.PCC	23	10	−45	25
**s3**	Left anterior cingulate cortex	L.ACC	32	−10	45	10
**s4**	Right anterior cingulate cortex	R.ACC	32	10	45	10
**s5**	Left inferior parietal lobule	L.IPL	40	−55	−55	20
**s6**	Right inferior parietal lobule	R.IPL	40	55	−55	20
**s7**	Left prefrontal cortex	L.PFC	46	−45	25	25
**s8**	Right prefrontal cortex	R.PFC	46	45	25	25
**s9**	Left lateral temporal cortex	L.LTC	21	−55	−15	−20
**s10**	Right lateral temporal cortex	R.LTC	21	55	−15	−20
**s11**	Left parahippocampal gyrus	L.PHG	36	−25	−30	−20
**s12**	Right parahippocampal gyrus	R.PHG	36	25	−30	−20

**Table 3:** Random Forest (RF) importance parameters (mean minimal depth, number of nodes, number of trees, times a root, accuracy decrease, Gini decrease, and *p*-value) and the direction of significance for the top significant variables (*p* < 0.05) are shown. All significant variables that were important to classify individuals into either the alcohol use disorder (AUD) group or the control (CTL) group have been listed. The order of these listed variables is based on *p*-values.

Variable	Mean Minimal Depth	No. of Nodes	No. of Trees	Times a Root	Accuracy Decrease	Gini Decrease	*p* Value	Direction
BIS_Total	1.0000	118	115	50	0.0265	2.0285	<0.0000	AUD > CTL
BIS_NP	1.4343	114	110	45	0.0156	1.4792	<0.0000	AUD > CTL
BIS_MI	1.6723	110	106	27	0.0117	1.4611	<0.0000	AUD > CTL
s1–s5 (L.PCC–L.IPL)	2.0023	103	101	27	0.0062	1.0920	<0.0000	AUD > CTL
s4–s8 (R.ACC–R.PFC)	1.7829	100	98	33	0.0051	1.2818	<0.0000	AUD > CTL
s3–s7 (L.ACC–L.PFC)	2.3930	75	74	25	0.0080	0.9634	<0.0000	AUD > CTL
s2–s4 (R.PCC–R.ACC)	2.9542	72	68	7	0.0030	0.6109	<0.0000	CTL > AUD
BIS_AI	2.9277	64	63	18	0.0042	0.6316	0.0019	AUD > CTL
s1–s8 (L.PCC–R.PFC)	3.1205	63	61	8	0.0004	0.5023	0.0029	CTL > AUD
s6–s9 (R.IPL–L.LTC)	3.0683	62	61	9	0.0039	0.5994	0.0044	CTL > AUD
Span_Fw (VST)	2.7308	62	59	29	0.0059	0.7670	0.0044	CTL > AUD
TotCor_Fw (VST)	2.7656	62	59	24	0.0083	0.7481	0.0044	CTL > AUD
s1–s4 (L.PCC–R.ACC)	2.8648	61	60	16	0.0022	0.6903	0.0065	CTL > AUD
s2–s5 (R.PCC–L.IPL)	3.1569	61	59	13	-0.0006	0.5276	0.0065	AUD > CTL
s3–s9 (L.ACC–L.LTC)	3.1359	61	58	12	0.0007	0.5459	0.0065	CTL > AUD
s4–s9 (R.ACC–L.LTC)	3.1395	61	59	5	0.0032	0.4423	0.0065	CTL > AUD
s8–s10 (R.PFC–R.LTC)	3.1983	57	56	13	-0.0009	0.5901	0.0268	CTL > AUD
s3–s4 (L.ACC–R.ACC)	3.3493	56	52	3	0.0007	0.3313	0.0368	AUD > CTL
s5–s8 (L.IPL–R.PFC)	3.3390	56	54	3	0.0003	0.3792	0.0368	CTL > AUD
s7–s10 (L.PFC–R.LTC)	3.2817	56	55	7	0.0022	0.4746	0.0368	CTL > AUD

L–Left; R–Right; PCC–Posterior cingulate cortex; ACC–Anterior cingulate cortex; IPL–Inferior parietal lobule; PFC–Prefrontal cortex; LTC–Lateral temporal cortex; VST–Visual Span Test.

**Table 4:** Correlations between the age of the participant and the significant variables in each group and the total sample.

Variable	AUD		CTL		ALL ^§^	
	r	*p*	r	*p*	r	*p*
BIS_AI	0.1006	0.5970	−0.1371	0.4699	0.0196	0.8829
BIS_MI	0.2346	0.2121	0.1156	0.5432	0.1993	0.1302
BIS_NP	0.0255	0.8936	0.2104	0.2644	0.0926	0.4854
BIS_Tot	0.1389	0.4643	0.1060	0.5772	0.1274	0.3363
s1–s4 (L.PCC–R.ACC)	−0.2429	0.1958	−0.1915	0.3107	−0.2262	0.0849
s1–s5 (L.PCC–L.IPL)	−0.1380	0.4669	−0.0108	0.9547	−0.0910	0.4932
s1–s8 (L.PCC–R.PFC)	−0.2273	0.2270	0.0488	0.7979	−0.1245	0.3475
s2–s4 (R.PCC–R.ACC)	−0.2834	0.1291	−0.1491	0.4315	−0.2317	0.0774
s2–s5 (R.PCC–L.IPL)	−0.1836	0.3316	−0.0677	0.7223	−0.1314	0.3212
s3–s4 (L.ACC–R.ACC)	−0.2658	0.1557	0.3790	0.0389 *	−0.0914	0.4910
s3–s7 (L.ACC–L.PFC)	0.0033	0.9860	0.0227	0.9051	0.0098	0.9415
s3–s9 (L.ACC–L.LTC)	0.2421	0.1974	0.1860	0.3251	0.2232	0.0892
s4–s8 (R.ACC–R.PFC)	0.0242	0.8991	−0.0323	0.8655	0.0049	0.9703
s4–s9 (R.ACC–L.LTC)	−0.0495	0.7952	0.1276	0.5016	0.0072	0.9570
s5–s8 (L.IPL–R.PFC)	−0.2007	0.2876	0.1877	0.3206	−0.0850	0.5222
s6–s9 (R.IPL–L.LTC)	−0.3233	0.0814	−0.2604	0.1646	−0.2956	0.0230 *
s7–s10 (L.PFC–R.LTC)	−0.1732	0.3602	0.1974	0.2957	−0.0629	0.6362
s8–s10 (R.PFC–R.LTC)	−0.0745	0.6957	−0.1071	0.5732	−0.0836	0.5290
Span_Fw (VST)	−0.3938	0.0313 *	−0.0429	0.8219	−0.2393	0.0679
TotCor_Fw (VST)	−0.4178	0.0216 *	−0.0231	0.9038	−0.2383	0.0692

L–Left; R–Right; PCC–Posterior cingulate cortex; ACC–Anterior cingulate cortex; IPL–Inferior parietal lobule; PFC–Prefrontal cortex; LTC–Lateral temporal cortex; VST–Visual Span Test. (*, *p* < 0.05; ^§^, Based on partial correlation adjusted for group effect).

## Appendix A

**Table A1:** Concepts and parameters used in Random Forest classification method.

**Trees:** Decision trees whose results are aggregated into one final result for classifying the factors or outcomes. Each tree is constructed based on a random (bootstrapped) subsample of the observations.
**Node:** A point in a tree, where a split occurs as a result of a ‘test’ on an attribute leading to binary outcomes (e.g., whether a coin flip results in head or tail). A binary split at a node partitions the data from the parent node into two daughter nodes.
**Branch:** The outcome of the test resulting in a split or two branches in a classification tree.
**Leaf:** A terminal node that has no children or branches.
**Random Forest ensemble:** Aggregation of individual decision trees in order to combine predictions (votes) from each tree. The class/group/outcome with most votes becomes the RF model’s prediction.
**Bagging:** It’s the short form of ‘bootstrap aggregating’, which is a method to improve classification by combining classifications of randomly generated training sets.
**Out of bag (OOB) estimate:** The observations that are not part of the bootstrap subsample are referred to as out-of-bag (OOB) observations. The OOB error refers to the classification error based on this subsample and serves as a validation of Random Forest model accuracy.
**Gini (mean) decrease:** It represents the importance of a specific feature/predictor/variable *(Vi)* for the classification or prediction. It’s the mean decrease in node impurity (classification error) of *Vi*. A higher Gini decrease indicates higher variable importance for *Vi*.
**Accuracy decrease:** Mean decrease in prediction accuracy after *Vi* is not taken into account.
**Mean minimal depth:** It refers to the number of nodes along the shortest path from the root node down to the nearest leaf node. Smaller depth for the *Vi* indicates its higher importance.
**Mtry:** A preset number of features/variables/predictors randomly selected (from the entire list) for splitting at each node in the construction of each decision tree.
**ntree:** A preset total number of trees to grow for a given model. Larger ‘ntree’ normally produce more stable models and more reliable predictions.
**Number of nodes:** Total number of nodes that use *Vi* for splitting (it is usually equal to number of trees if trees are shallow).
**Times a root:** Total number of trees in which *Vi* is used for splitting the root node (i.e., the whole sample is divided into two based on the value of *Vi*).
**p value:** probability value of hypothesis testing based on a one-sided binomial test that indicates whether the observed number of successes (number of nodes in which *Vi* was used for splitting) exceeds the theoretical number of successes if they were random.
